# Internal Relative Humidity, Autogenous Shrinkage, and Strength of Cement Mortar Modified with Superabsorbent Polymers

**DOI:** 10.3390/polym10101074

**Published:** 2018-09-28

**Authors:** Girum Urgessa, Ki-Bong Choi, Jung Heum Yeon

**Affiliations:** 1 Sid and Reva Dewberry Department of Civil, Environmental, and Infrastructure Engineering, George Mason University, Fairfax, VA 22030, USA; gurgessa@gmu.edu; 2 Department of Architectural Engineering, Gachon University, Seongnam 13120, Korea; kbchoi@gachon.ac.kr; 3 Department of Civil and Environmental Engineering, Gachon University, Seongnam 13120, Korea

**Keywords:** superabsorbent polymers, internal curing, autogenous shrinkage, internal relative humidity, coefficient of thermal expansion, strength

## Abstract

Laboratory evaluations were performed to investigate the effect of internal curing (IC) by superabsorbent polymers (SAP) on the internal relative humidity (IRH), autogenous shrinkage, coefficient of thermal expansion (CTE), and strength characteristics of low water-cement ratio (*w*/*c*) mortars. Four types of SAP with different cross-linking densities and particle sizes were used. Test results showed that the SAP inclusion effectively mitigated the IRH drops due to self-desiccation and corresponding autogenous shrinkage, and the IC effectiveness tended to increase with an increased SAP dosage. The greater the cross-linking density and particle size of SAP, the less the IRH drop and autogenous shrinkage. The trend of autogenous shrinkage developments was in good agreement with that of IRH changes, with nearly linear relationships between them. Both immediate deformation (ID)-based and full response-based CTEs were rarely affected by SAP inclusions. There were no substantial losses in compressive and flexural strengths of SAP-modified mortar compared to reference plain mortar. The findings revealed that SAPs can be effectively used to reduce the shrinkage cracking potential of low *w*/*c* cement-based materials at early ages, without compromising mechanical and thermal characteristics.

## 1. Introduction

High-performance concrete (HPC), which typically has a low water-cementitious ratio (*w*/*cm*), is prone to early-age shrinkage cracking induced by internal drying [1,2]. The internal drying is a process that consumes water from within the concrete itself (i.e., water from pores) for delayed hydration of unhydrated cement grains present in the matrix [2,3]. For this reason, the internal drying is clearly distinguished from typical long-term drying triggered by “diffusion-evaporation” mechanisms under prolonged environmental exposure. The internal drying is also often termed self-desiccation, and this propensity consequently increases the water molecule-holding capacity at the meniscus between liquid water and vapor in capillary pores, reducing the internal relative humidity (IRH) of a cementitious material system [4,5,6,7]. The bulk strain caused by the IRH changes under sealed, isothermal, and stress-free conditions is referred to as autogenous shrinkage [2,3,7], which is considered a crucial factor in raising the probability of early-age non-structural cracking in modern concrete members.

A variety of research efforts are underway to develop efficient strategies to tackle autogenous shrinkage in cement-based materials. Taylor [8] and Nakataki and Gomi [9] described that shrinkage-compensating expansive cement leads to volume expansion of concrete after initial setting via ettringite formation, thereby offsetting subsequent volume reduction due to shrinkage. Another study by Folliard [10] confirmed that shrinkage-reducing admixtures (SRA) can significantly reduce the shrinkage and resulting restrained shrinkage cracking potential by relieving the surface tension of pore fluid. Swayze [11] attempted to compensate autogenous shrinkage using early-age thermal expansion and mechanical properties enhancement by taking advantage of heat of hydration of cement. The use of stiffer aggregates was suggested to control the magnitude of shrinkage by Neville and Brooks [12]. Supplying extra water from the surroundings through the surface via fog misting and water ponding may also be used during initial curing, but the effectiveness of curing water ingress from the surroundings may be quite limited when it comes to HPC, because the capillary porosity in low-*w*/*cm* concrete undergoes early depercolation [13]. As an alternative to overcoming such practical limitations, providing extra curing water from the internal reservoirs by means of incorporation of highly absorptive substances (referred to as “internal curing (IC)”), particularly lightweight aggregate (LWA) [14] and superabsorbent polymers (SAP), has been gaining increasing attention as the new concept of autogenous shrinkage mitigation strategy. In addition to the shrinkage mitigating capability, the IC allows concrete to further develop potential properties by promoting the degree of hydration and thus is becoming one of the promising treatment options for HPC curing.

SAP is a cross-linked polymeric substance that can momentarily absorb a substantial amount of water or aqueous solutions (up to 500 g within a couple of minutes). In the concrete industry, since Jensen and Hansen initiated a pioneering work on “water-entrained concrete” using SAP [15,16], numerous follow-up studies have been done in the last few decades to seek potential possible uses of SAPs as an internal curing agent for cement-based materials, for instance, autogenous shrinkage mitigation [16,17,18,19], later-age mechanical properties improvement [20,21,22,23], rheology modification [24,25,26], self-sealing and healing capability [27,28,29,30,31], shrinkage crack control [20,32], coefficient of thermal expansion (CTE) control [33], frost damage mitigation [34,35], and absorption and desorption kinetics under alkaline environment [17,36,37]. The efficiency of IC is closely concerned with the absorption and desorption kinetics of SAP. If SAP absorbs mixing water quickly or releases absorbed water slowly, the autogenous shrinkage may not be effectively controlled due to the shortage of curing water supply. On the contrary, if SAP absorbs water slowly or releases part of absorbed water early (prior to initial setting), the amount of water serving as free water increases (*w*/*c* increases), which may adversely affect the mechanical properties and durability. The goals of the present study are to investigate the effects of SAPs with different cross-linking densities and particle sizes on the properties of low *w*/*c* mortars, including autogenous shrinkage and IRH. In addition, the CTE and strength characteristics of SAP-modified mortar are assessed.

## 2. Materials and Methods

### 2.1. Materials

ASTM C150 Type I Portland cement was used in this study. The chemical composition and physical properties of the cement are summarized in Table 1. Standard sand conforming to KS F 2406 was used as fine aggregate. The fine aggregate had a specific gravity of 2.65, a fineness modulus (FM) of 2.87, an absorption capacity of 1.02%, a SiO_2_ content of 98.4%, and a median diameter (D50) of 533 μm. A lignosulfonate-based water reducer was used at a recommended dosage of 5 mL/cwt.

**Figure 1:**
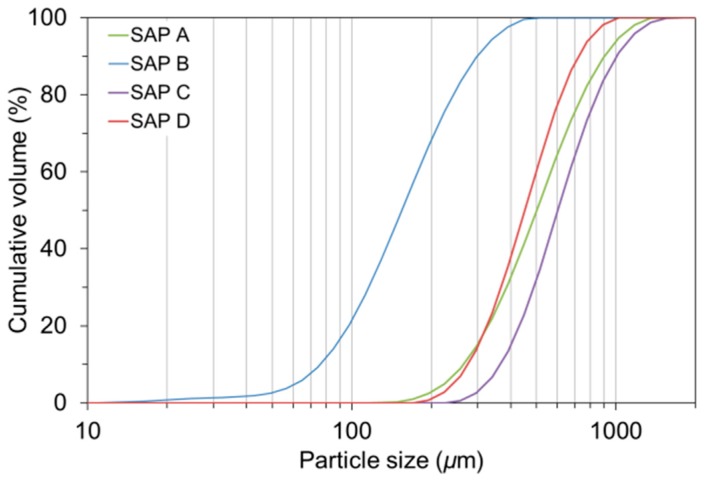
Cumulative particle size distributions of SAPs used.

**Figure 2:**
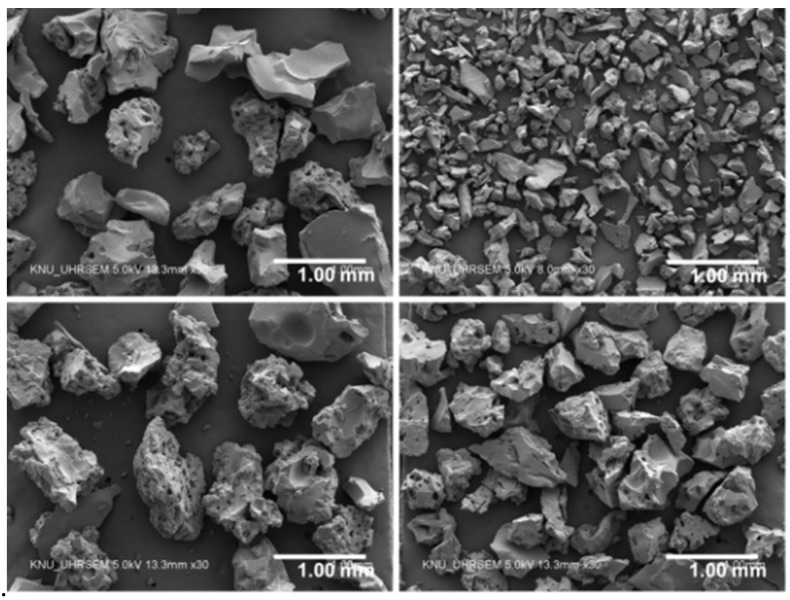
SEM images of SAP A, SAP B, SAP C, and SAP D (clockwise from top left).

Four commercially available sodium polyacrylate SAPs were used as an IC agent. The SAPs were produced by bulk polymerization first, followed by pulverization into fine powder. Table 2 shows the chemical and physical properties of the SAPs used. The SAPs with three different levels of cross-linking density and four different levels of particle size were used as shown in Table 3. The cumulative volume versus particle size distribution measured by means of a particle size analyzer (Hydro 2000S; Malvern Instruments, Malvern, UK) is presented in Figure 1. The mean dry particle size was found to be 535.0 μm for SAP A, 165.8 μm for SAP B, 644.8 μm for SAP C, and 482.4 μm for SAP D. Figure 2 shows the microscopic images of SAPs obtained using an ultra-high-resolution scanning electron microscope (UHR-SEM) (Hitachi S-4800; Hitachi, Tokyo, Japan).

The mixture proportions for plain and SAP-modified mortars used in this study are shown in Table 4. Three different levels of SAPs (i.e., 0.2%, 0.4%, and 0.6% of cement by weight) were used for SAP D. The total water content of each mixture was adjusted per the absorption capacities of SAPs measured under mixing condition so as to keep the workability comparable with each other [37]. All the mixtures were fabricated at an effective water-cement ratio (*w*/*c*) of 0.30 and a fine aggregate-to-cement ratio of 2.75. The flow value of all mixtures was consistent with 120 ± 3 mm. The factorial experiment was designed to address the effects of both SAP dosage (SAP D 0.2, SAP D 0.4, and SAP D 0.6) and type (SAP A 0.4, SAP B 0.4, SAP C 0.4, and SAP D 0.4).

### 2.2. Test Methods

#### 2.2.1. Autogenous Shrinkage and IRH Measurements

**Figure 3:**
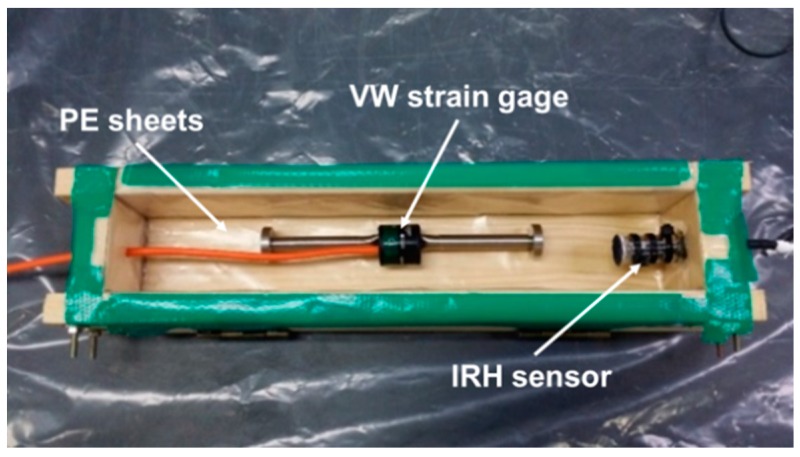
Experimental setup for IRH and autogenous shrinkage.

A linear autogenous shrinkage evolution was measured using a 50 × 50 × 300 mm^3^ prismatic specimen for 50 days after final setting. The final set time was determined based on ASTM C403 (Standard Test Method for Time of Setting of Concrete Mixtures by Penetration Resistance). An embedment-type vibrating wire strain gage (VWSG) (Model 1240; ACE Instrument Co., Ltd., Gunpo, Korea) with a range of 3000 με, a gage length of 153 mm, and a resolution of 0.5 με was installed at the center of the cross-section along the longitudinal axis. The VWSG was equipped with a built-in thermistor for thermal compensation. For IRH measurements, a capacitive humidity sensor (HYT-939; IST AG, Ebnat-Kappel, Switzerland) with an accuracy of ±1.8% RH and ±0.2 °C, and a 16-channel data logger (MSHTDL-16, Bientron, Incheon, Korea) were used. The IRH sensor was treated with an engineered membrane that allows water vapor pass through before it was situated in the mold. Figure 3 shows the experimental setup for IRH and autogenous shrinkage measurements before casting fresh mortar.

**Figure 4:**
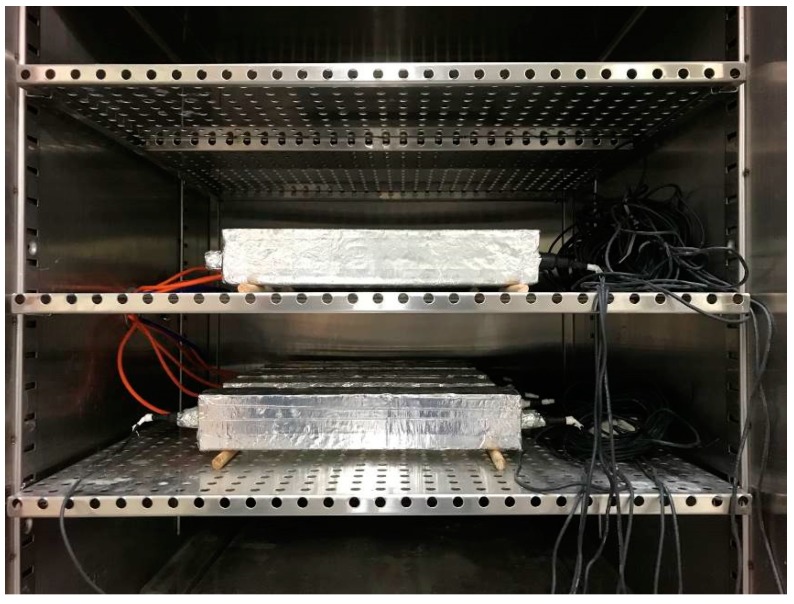
Testing in progress in environmental chamber.

Double layers of a polyethylene (PE) thin film were placed onto every inner surface of the mold so that the friction effect prior to demolding could be eliminated. Fresh mortar was then placed in two layers, and each layer was vibrated using a table-type vibrator for 60 s after manual compaction in order to ensure proper compaction. After 24 h of casting, the specimens were demolded and then promptly sealed with adhesive-backed aluminum foil so as to prevent a moisture loss due to evaporation. Subsequently, the fabricated specimens were stored in an environmental chamber that maintains 23 ± 1 °C and 75% relative humidity (RH) while collecting data using a data logger (CR1000; Campbell Scientific, Inc., Logan, UT, USA), as shown in Figure 4. Two rollers were placed underneath the specimen to allow free length changes during the test.

#### 2.2.2. Coefficient of Thermal Expansion Test

**Figure 5:**
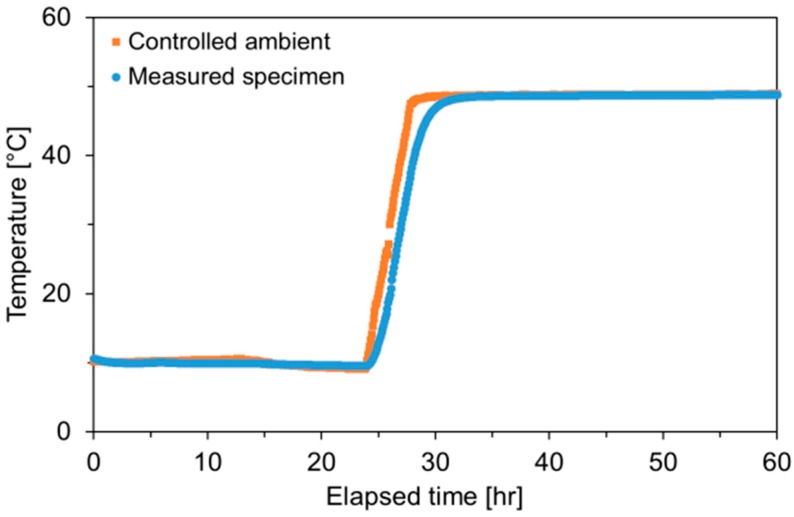
Ambient and specimen temperature histories measured during CTE test.

Once the autogenous shrinkage and IRH tests were completed, the specimens were unwrapped and stored in a controlled environment of 20 ± 1 °C and 70% RH until the specimens achieve an IRH of 70 ± 5%, where the maximum CTE occurs [4,5,6]. The RH values at the time of CTE testing were: 69.29% for Plain, 75.07% for SAP A 0.4, 68.12% for SAP B 0.4, 74.25% for SAP C 0.4, 67.09% SAP D 0.2, 71.88% for SAP D 0.4, and 73.26% for SAP D 0.6. This step was essential, to eliminate the additional effect of IRH changes induced by temperature changes on the CTE (i.e., hygrothermic effects) [4,6,38]. As soon as the target RH range was reached, the specimens were tightly sealed again using aluminum foil tape and then stabilized at 20 ± 1 °C for 6 weeks to obtain IRH equilibrium within the specimens [4]. Subsequently, thermal strain was measured for the temperature change from 10 to 50 °C based on AASHTO TP-60 [39] with a heating rate of 6.7 °C/h. For this procedure, an air circulating environmental chamber was used to minimize the chances of moisture exchange between the specimen and surrounding water that might occur when water was used as the medium. Once the specimens reached 50 °C, the temperature was held constant for >30 h to ensure thermal equilibrium. Based on the strain changes caused by the temperature rise, CTE was determined. Figure 5 shows both controlled ambient and specimen temperature histories measured during the CTE testing.

#### 2.2.3. Flexural and Compressive Strength Tests

Flexural and compressive strengths were measured as per ASTM C348 (Standard Test Method for Flexural Strength of Hydraulic-Cement Mortars) and ASTM C349 (Standard Method for Compressive Strength of Hydraulic-Cement Mortars Using Portions of Prisms Broken in Flexure), respectively. Specimens were cured in an environmental chamber maintaining 23 °C and 95% RH after casting. After the 24-h curing period, the specimens were demolded and stored in double layers of air-tight sealable bags for moisture retention. For flexural strength testing, three replicate specimens with dimensions of 40 × 40 × 160 mm^3^ were loaded by center point loading with a load rate of 50 N/s at the ages of 1, 3, 7, and 28 days. In addition, compressive strength was measured using the six broken portions in a load control scheme (2400 N/s) at the same ages. A 200-kN capacity universal testing machine (Instron 8502; Instron, Norwood, MA, USA) was consistently used to apply loading.

## 3. Results and Discussion

### 3.1. IRH Changes and Autogenous Shrinkage

**Figure 6:**
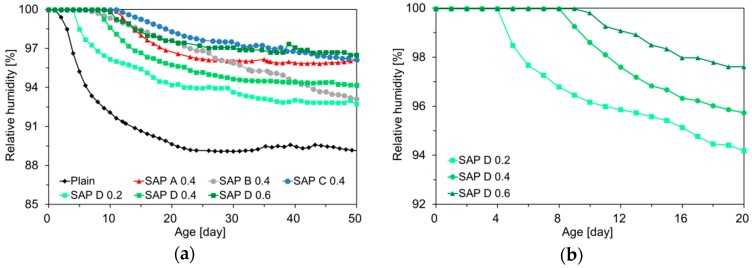
Changes in IRH over time: (**a**) 50 days for all specimens; (**b**) first 20 days only for SAP D series.

Figure 6 shows the result of IRH measurements for plain and various SAP-modified mixtures. Please note that the IRH gradually decreased over time due to the internal water consumption induced by self-desiccation. It was also found that the SAP-modified mixtures maintained a higher level of IRH than the plain mixture throughout the measurement period, which was well in line with the results of previous studies [17,19,36,40]. This is because the SAPs distributed across the mortar volume supplied extra curing water to the hydrating matrix, whereas the plain mixture had no internal source for additional water to replenish the emptying capillary pores.

The propensity of IRH drop was somewhat different depending on the type and dosage of SAP. It was estimated from the result of SAP D series (SAP D 0.2, SAP D 0.4, and SAP D 0.6) that the 50-day IRH drops for SAP D 0.2, SAP D 0.4, and SAP D 0.6 were 66.8%, 53.6%, and 32.2% of that for reference mortar, respectively. Moreover, the results revealed that, while the plain mixture commenced abrupt internal drying soon after final setting, the IRH started to drop at about 4 days for SAP D 0.2, 8 days for SAP D 0.4, and 10 days for SAP D 0.6. This is because the specimen with a greater SAP addition released the IC water for a prolonged period, which compensated and delayed the subsequent self-desiccation [19,41].

The result of 0.4% addition series (SAP A 0.4, SAP B 0.4, SAP C 0.4, and SAP D 0.4) showed some discrepancies in IRH behavior depending on the type (particle size and cross-linking density) of SAPs. First, it can be seen that SAP C, which has the greatest cross-linking density and largest particle size, maintained the highest IRH profile over the measurement period. The most probable reason is that the more numerous the cross-links between polymer chains, the higher the elastic retraction forces are applied to the network structure, rapidly squeezing out the water absorbed in SAP to the matrix [37]. On the contrary, SAP D, having a medium grain size and cross-linking density, exhibited the greatest IRH drop during the first 50 days. This is attributed to the high absorption capacity (10.99 g/g dry SAP) and medium elastic retraction forces of SAP D, which made the SAP particles retain a large amount of IC water for a relatively longer period [37]. It is also noteworthy that SAP B, having the finest particle size, showed an almost linear IRH drop over time, which is quite similar to the desorption kinetics of SAP B evaluated in the previous study [37]. The shorter water release time appears to be mainly associated with the larger specific surface area and small nucleus of SAP B. Also, the weaker intermolecular attraction forces (van der Waals forces) acted on the small particles might contribute to the rapid and steady losses of absorbed water [42].

**Figure 7:**
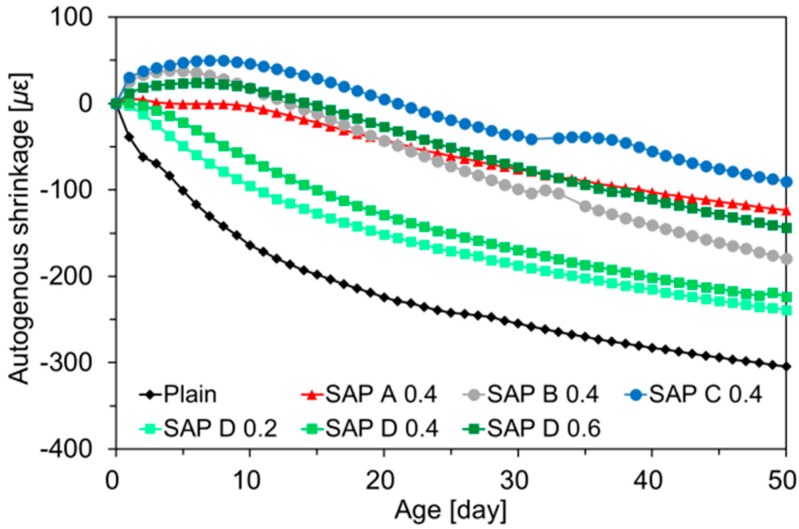
Autogenous shrinkage developments.

Figure 7 depicts the autogenous shrinkage evolutions monitored in the specimens identical with those for IRH measurements. Please note that the trend of autogenous shrinkage evolutions was quite similar to that of IRH changes presented in Figure 6, similarly with the result of Wang et al. [43] and Lura et al. [7]. This is because the mortar shrank in response to the increased surface tension in capillaries induced by the IRH drop. The inclusion of SAP tended to effectively mitigate the autogenous shrinkage. Indeed, in some SAP-modified mixtures, swelling of up to about 60 με was observed at early ages, compensating for the subsequent shrinkage. In the case of SAP C 0.4, which had the greatest cross-linking density and largest particle size, the autogenous shrinkage at 50 days was found to be 90 με, which was approximately 70% lower than that of plain mixture (i.e., 304 με). Interestingly, the effects of SAP type and addition level on the autogenous shrinkage were also nearly identical to those on the IRH behavior.

**Figure 8:**
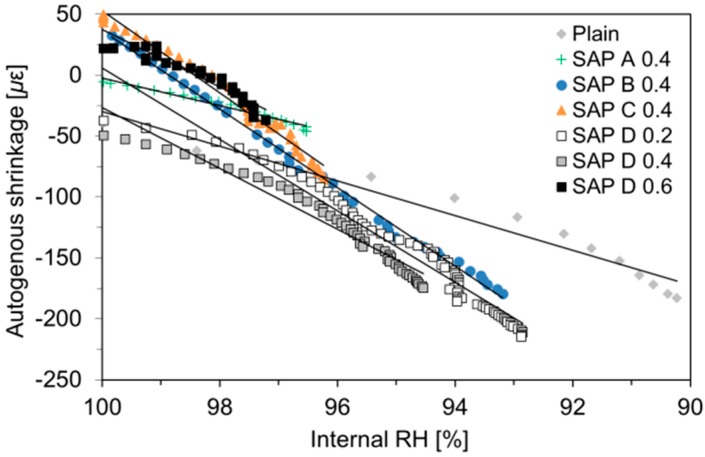
Relationships between measured IRH and autogenous shrinkage.

The relationships between the measured IRH and autogenous shrinkage are plotted in Figure 8, along with the linear fitting equations for each specimen. Herein, the characteristic value correlating the unit IRH drop to autogenous shrinkage is defined as a hygral coefficient of autogenous shrinkage, which was calculated as follows [44]:

(1) $$\alpha^{sh}=\frac{\Delta \varepsilon_{i}{}^{sh}}{\Delta RH_{i}}$$

where *α^sh^* is the hygral coefficient of autogenous shrinkage [με/%RH]; ∆*ε_i_^sh^* is the increment of autogenous shrinkage during the *i*th time step [με]; and ∆*RH_i_* is the IRH change during the *i*th time step (%RH).

The analysis results show that there were almost linear correlations between the IRH and autogenous shrinkage with a high coefficient of determination (*R*^2^) of 0.899 to 0.997, indicating that a statistically meaningful linear relationship existed between the IRH and autogenous shrinkage. The hygral coefficient of autogenous shrinkage evaluated in this study was found to vary from 11.3 to 33.7 με/%RH, which was in good agreement with the results obtained by Wang et al. (8–21 με/%RH) [45] and Lura et al. (approximately 20 με/%RH) [7]. It can be observed that the SAP-modified mixtures had a much greater hygral coefficient of autogenous shrinkage than the plain mixture. This is most likely because the SAP-modified mortars generally have lower modulus than plain mortar due to the inclusions of SAP, which is much softer than the hydrated matrix [46]. In addition, the test results indicated that: (1) the hygral coefficient of autogenous shrinkage slightly decreased as the SAP addition increased (from SAP D series); and (2) the hygral coefficient of autogenous shrinkage was moderately proportional to the cross-linking density of SAP (from 0.4% series).

### 3.2. Coefficient of Thermal Expansion

Two thermal dilation components—immediate deformation (ID) and delayed deformation (DD)—were considered for CTE evaluations. While the ID is defined as an instantaneous thermal deformation occurring simultaneously with temperature changes, the DD is a time-dependent thermal deformation taking place after thermal equilibrium. Owing to the complexity of the principles behind the thermal dilation of cement-based materials, different approaches have been adopted in defining CTE of cement-based materials. While Meyers [47], Sellevold and Bjøntegaard [6] and Kada et al. [48] attempted to evaluate the CTE solely based on the ID, since the full response includes both pure thermal dilation and additional time-dependent deformation resulting from moisture diffusion, Bazant [38], Grasley and Lange [5], Cusson and Hoogeveen [49], and Yeon et al. [4] determined the CTE based on the total deformation, including a delayed component. In this study, the CTE was assessed on the basis of both ID and full thermal response.

**Figure 9:**
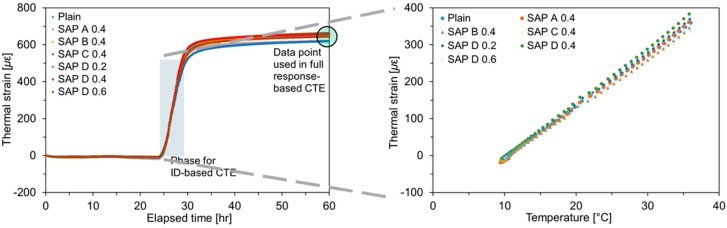
Results of CTE test: thermal strain responses induced by imposed temperature rise (**left**); relationships between thermal strain and temperature for determining ID-based CTE (**right**).

The thermal strain histories measured for all specimens are given in Figure 9. The thermal strain abruptly increased in response to the imposed temperature history shown in Figure 6. The obtained thermal strain curves well coincided with the thermal response of partially saturated (i.e., 70% RH) cement-based materials theoretically evidenced by Bazant [38], without subsequent contraction caused by the gradual flow of expanding water out of the pores during the isothermal hold. Figure 9 also includes the schematics of how the ID- and full response-based CTEs were calculated in this study. While the ID-based CTE was evaluated by linearly fitting the temperature versus thermal strain curve during the heating phase, as shown in the right figure [50], the full response-based CTE was based on the ultimate thermal strain at the end of temperature step as indicated in the left figure [4].

**Figure 10:**
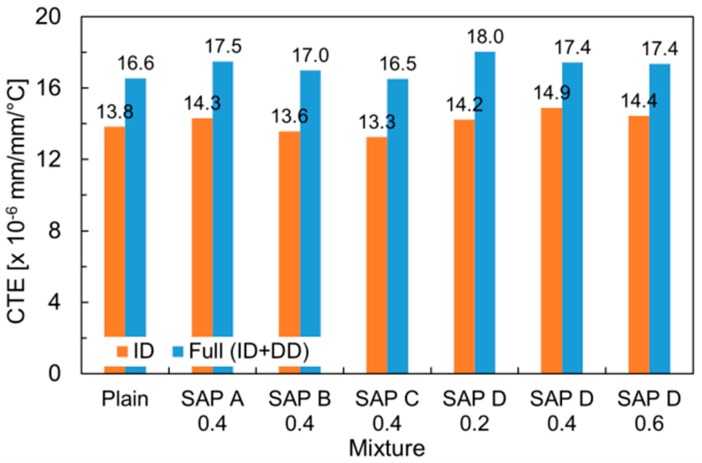
Result of ID- and full response-based CTE measurements.

The CTE values evaluated based on the temperature input and resulting thermal strain are summarized in Figure 10. The CTE values ranged between 13.3 to 14.9 με/°C, which were slightly higher than the normal CTE value for cement mortar (i.e., 10.5 to 13.5 με/°C). This was because the present study evaluated the CTE at the intermediate IRH level (i.e., 70% ± 5%), where the hygrothermic reaction mainly governs thermal volume changes. It can also be seen that the CTEs evaluated based on the full response (ID + DD) were all greater than those based on the ID only because, as mentioned above, no contraction took place after holding the final temperature at 50 °C. It is interesting to note that the ID- and full response-based CTEs exhibited quite similar trends with each other, with a 3.1 με/°C shift on average.

The SAP addition was found to have insignificant effects on the CTE as there were no remarkable discrepancies among the mixtures. The average percentage difference of the ID- and full response-based CTEs with respect to the reference mortar was found to be only 3.5% and 4.0%, respectively. The reason for this result is that while the pore size that substantially affects the thermal dilation of cement-based materials ranges from 0.1 to 10 μm in diameter (medium to macro capillaries), the pore diameter entrained by swollen SAPs is mainly distributed between 200 to 500 μm [51], yielding minimal influences on the thermal dilation. No consistent trends were found between the measured CTEs and the physical and chemical characteristics (i.e., cross-linking density, particle size, and absorption capacity) and addition level of SAP.

### 3.3. Flexural and Compressive Strengths

**Figure 11:**
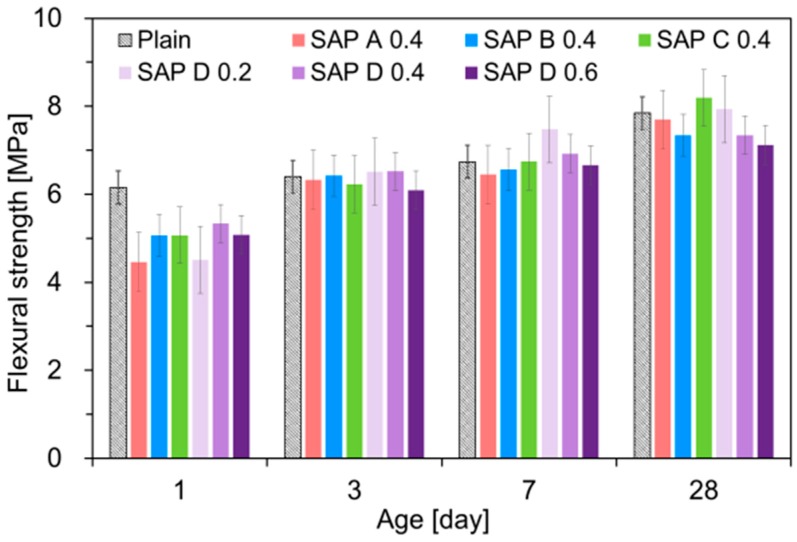
Result of flexural strength measurements.

**Figure 12:**
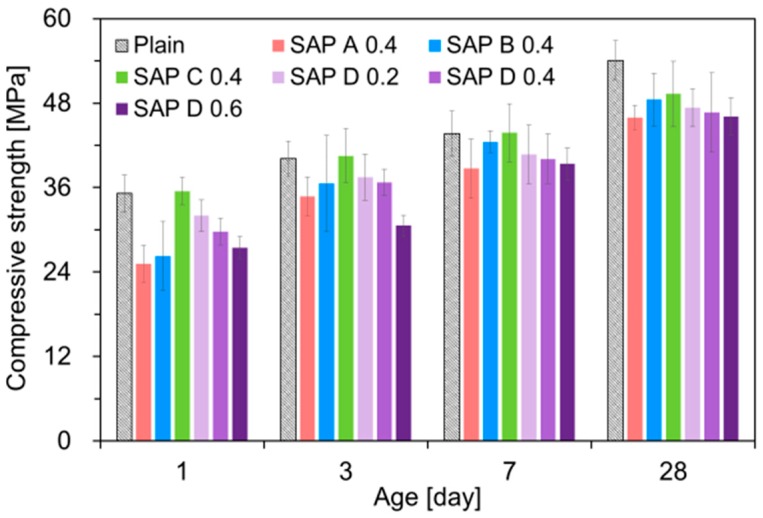
Result of compressive strength measurements.

Figure 11 and Figure 12 show the effects of SAP type and addition level on the flexural and compressive strengths, respectively. Overall, both flexural and compressive strengths were reduced with the inclusion of SAPs, similarly with the results of previous studies [20,22,23,37]. Particularly, the strength reductions were prominent at early ages. On average, the 1-day flexural and compressive strengths were reduced by up to 27.5% and 28.5%, respectively, when SAP was added. Even though the early-age strengths were noticeably lower for SAP-modified mixtures, the differences tended to become smaller with age for flexural strength; some SAP-modified mixtures started to exceed the strength of plain mixture after 3 days. This is because the extra water released from SAPs provided an IC effect, which promoted the degree of hydration of cement [52].

It can be seen from the SAP D series that, even with the same effective *w*/*c* of 0.30, both flexural and compressive strengths tended to monotonically decrease as the SAP addition increased, which were in good agreement with the results of previous studies [20,22,23,37]. The strength reductions were primarily attributed to the increased macro-porosity created by the contraction of swollen SAPs upon water release [23,35,53,54] and the reduced load-bearing capacity resulting from SAP inclusions, whose stiffness is much lower than that of hydrated matrix [46]. Furthermore, the strength losses may arise due to the “angular” pores acting as stress risers formed by the swelling of irregular-shape SAP grains [23] and delayed final setting time with increased SAP additions: 6.3 h for plain, 7.8 h for SAP D 0.2, 8.7 h for SAP D 0.4, and 11.9 h for SAP D 0.6.

Relatively higher strengths were observed for SAP C. This finding well matches the IRH behavior given in Figure 6, in which the IRH was kept higher than other mixtures throughout the entire measurement period. This indicates that the SAP with better moisture retention performance may be beneficial for strength gains. On the other hand, the strength reductions became more pronounced with the SAPs with a high absorptivity (SAP A and SAP D), which was in good agreement with the result of Klemm et al. [55].

## 4. Concluding Remarks

This study investigated the effect of internal curing (IC) by superabsorbent polymers (SAP) on the internal relative humidity (IRH) and autogenous shrinkage of mortar with a low water-cement ratio (*w*/*c*). The coefficient of thermal expansion (CTE) and strength characteristics of SAP-modified mortar were also examined. To gain a comprehensive understanding of the SAP influences, performance of four SAPs with different cross-linking densities and particle sizes were evaluated. From the findings of this study, the following conclusions can be made:

- The inclusion of SAPs can be a viable option to alleviate IRH drops and corresponding autogenous shrinkage developments in cementitious composites with a low *w*/*c*.
- The effectiveness of autogenous shrinkage mitigation became greater as the dosage of the SAP increased.
- SAPs with higher cross-linking density and larger particle size worked more effectively in mitigating autogenous shrinkage.
- The trend of autogenous shrinkage was quite close to that of IRH changes. The autogenous shrinkage was almost a linear function of IRH with a coefficient of determination (*R*^2^) of more than 0.90.
- SAP additions had insignificant effects on both immediate deformation (ID)-based- and full response-based CTEs.
- SAP inclusions had minimal adverse effects on the flexural strength characteristics of low *w*/*c* mortars although the compressive strength was moderately reduced at all ages. Particularly for flexural strength, the SAP with greater moisture retention capacity resulted in even higher later-age strength gains than the plain mixture.

This research mainly highlighted the effectiveness of SAP inclusion on the autogenous shrinkage mitigation in cement mortar with a low *w*/*c*. Further comprehensive studies are required to analyze how the SAP inclusion affects the actual shrinkage stress evolution and resulting cracking potential when the autogenous shrinkage is combined with creep, modulus of elasticity, and tensile strength characteristics under restrained condition.

## Figures and Tables

**Table 1:** Chemical composition and physical properties of cement.

Chemical Composition (%)								Fineness (m^2^/kg)	Specific Gravity
SiO_2_	Al_2_O_3_	Fe_2_O_3_	CaO	MgO	SO_3_	K_2_O	Na_2_O		
19.7	5.33	2.90	61.5	3.81	2.54	0.86	0.18	370	3.15

**Table 2:** Chemical and physical characteristics of SAPs.

Chemical Nomenclature	Chemical Formula	Molar Mass (g/mol)	Constitutional Formula	Density (g/cm^3^)	Appearance
Poly (sodium prop-2-enoate)	(C_3_H_3_NaO_2_)_n_	Variable	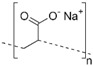	1.22	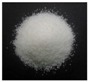

**Table 3:** General properties of SAPs used.

Type of SAP	Dry particle Size (Diameter)	Cross-Linking Density	Rate of Moisture Uptake [36]	Free Absorption Capacity (in Cement Filtrate at 60 min) [36]	Absorption Capacity under Mixing Condition [36]
SAP A	#80–20 mesh (177–841 μm)	Low	Low	Medium-high (38.91 g/g SAP)	High (12.70 g/g SAP)
SAP B	#120–80 mesh (125–177 μm)	Medium	High	Medium (35.43 g/g SAP)	Medium (8.75 g/g SAP)
SAP C	#80–20 mesh (177–841 μm)	High	Low	Low (31.01 g/g SAP)	Low (4.82 g/g SAP)
SAP D	#100–40 mesh (149–400 μm)	Medium	Medium	High (42.71 g/g SAP)	Medium-high (10.99 g/g SAP)

**Table 4:** Mixture proportions of SAP-modified mortar.

Mixture	Weight per Unit Volume (kg/m^3^)					
	Cement	Sand	Water	SAP	IC Water ^a^	Water Reducer
Plain	604.2	1661.6	181.3	-	-	3.02
SAP A 0.4	586.2	1612.1	175.9	2.35	29.8	2.93
SAP B 0.4	591.7	1627.2	177.5	2.37	20.7	2.96
SAP C 0.4	597.3	1642.6	179.2	2.39	11.5	2.99
SAP D 0.4	588.6	1618.7	176.6	2.35	25.9	2.94
SAP D 0.2	596.3	1639.8	178.9	1.19	13.1	2.98
SAP D 0.6	581.1	1598.0	174.3	3.49	38.3	2.91

^a^ Internal curing water: extra water supply for initial moisture uptake of dry SAPs.
